# Investigating the role of EGFR signalling in muscle dystrophies: implications for Duchenne muscular dystrophy

**DOI:** 10.1038/s41419-025-08193-9

**Published:** 2026-01-09

**Authors:** Esther Fernández-Simón, Ainoa Tejedera-Villafranca, Xiomara Fernández-Garabay, James Clark, Alexandra Monceau, Elisa Villalobos, Dan Cox, Javier Ramón Azcón, Juan M. Fernández-Costa, Jordi Diaz-Manera

**Affiliations:** 1 John Walton Muscular Dystrophy Research Centre, Institute of Genetic Medicine, Newcastle University, Newcastle upon Tyne, UK; 2 Institute for Bioengineering of Catalonia (IBEC), The Barcelona Institute of Science and Technology (BIST), Barcelona, Spain; 3 Institució Catalana de Recerca i Estudis Avançats (ICREA), Barcelona, Spain

**Keywords:** Molecular biology, Pathogenesis

## Abstract

The degeneration of the muscle in muscle dystrophies involves complex interactions among the different cell types. Here, we have used datasets from single-nuclei RNA sequencing (snRNAseq) of Duchenne Muscular Dystrophy (DMD) muscle samples to study the dysregulation of molecular pathways compared to healthy control muscle. We have observed that the epidermal growth factor (EGF) signaling is upregulated in DMD by an increase of the ligands EGF and EGF containing fibulin extracellular matrix protein 1 (EFEMP1). This study explores the role of EGF and EFEMP1 in FAPs and myoblasts in vitro. We provide evidence that EFEMP1 is secreted by FAPs in DMD and is mainly involved with increased myotube size but without enhancing muscle strength. Conversely, EGF enhances fibrotic differentiation in FAPs and promote smaller, proliferative myotubes in myoblasts, aligning with a fibrotic and dysfunctional muscle phenotype in DMD. The cellular differences from both ligands can be explained by the interactions with the receptor type, with EGF activating both EGFR and ErbB2, while EFEMP1 selectively maintained ErbB4 in an inactive state but promoting EGFR-ErbB2 and ErbB2-ErbB4 heterodimerization, potentially enhancing EGF signaling. Consequently, this study examinates the alteration of the EGF signalling in DMD and provides new molecular interactions in muscle that can be useful for targeted therapies of muscle degeneration.

## Introduction

Duchenne muscular dystrophy (DMD) is a genetic disease characterized by progressive degeneration of the skeletal muscle leading to permanent weakness and disability. Most patients die during the third decade of life due to cardiac or respiratory complications [1]. This debilitating condition is produced by pathogenic variants in the dystrophin gene, leading to the absence or severe reduction of dystrophin protein in muscle fibers [2]. As a result, the skeletal muscle of individuals with DMD undergoes a cascade of pathological changes consisting on muscle fiber injury and inflammation triggering repeated cycles of muscle regeneration and degeneration that ultimately fails, leading to loss of muscle fibers and their replacement by fibrofatty tissue [3]. These alterations ultimately compromise muscle function, contributing to the debilitating nature of the disease.

The process of muscle degeneration in DMD involves complex interactions among muscle cell types, particularly fibroadipogenic progenitor cells (FAPs) and myoblasts [4, 5]. Myoblasts drive muscle regeneration but face a microenvironment in DMD marked by chronic inflammation and fibrosis [6, 7]. FAPs contribute to either muscle regeneration or degeneration by promoting fibrosis and fat deposition [8, 9]. Despite considerable progress in the understanding of the degenerative process in DMD, there is still a considerable lack of knowledge of what are the cellular and molecular consequences of the muscle degeneration.

Single-nuclei RNA sequencing (snRNAseq) of early-stage DMD patient muscle samples compared to healthy controls revealed dysregulated gene expression in muscle fibers and FAPs. Results showed increased FAP proliferation, accompanied by elevated extracellular matrix (ECM) production in DMD. FAPs were the main source of ECM and secreted signals affecting various cell types, including muscle fibers, satellite cells, and inflammatory cells [10]. The epidermal growth factor (EGF) pathway was one of the most upregulated signaling pathways in DMD, prompting further investigation into its role in muscle regeneration. Based on this data we decided to validate these results and further investigate how EGF signaling influence the behavior of muscle resident cells key in the process of muscle regeneration.

The EGFR family consists of four receptor subtypes: EGFR/ErbB1/HER1, ErbB2/Neu/HER2, ErbB3/HER3, and ErbB4/HER4. Each subtype is activated by binding of a family of EGF-related peptides, such as EGF or transforming growth factor α (TGF-α) with different specificities [11]. EGFR activation triggers different signalling pathways that are involved in cell migration, proliferation, differentiation and survival [12, 13]. The activation of the pathway is ligand-dependent making different activation and downstream signaling according to the specific molecule bound to the receptor as well as the presence and abundance of the different EGFR receptors [14]. The role of EGFR signaling in muscle has not been fully addressed. Kim et al. studied the activation of EGFR in muscle from mice and found that constitutively signaling affected the skeletal muscle fiber type, negatively regulating the slow-twitch fibers [15]. The decrease of type I fibers increased the susceptibility to muscle mass loss and these effects were attenuated by pharmacologic inhibition of EGFR. Similar results were found by Ciano et al. who reported that EGFR signaling pathway is relevant in patients with chronic obstructive pulmonary disease contributing to the loss of slow-twitch fibers, while blocking EGFR signaling increased the number of slow-twitch fibers [16]. On the other hand, Wang et al. found that EGFR signaling in muscle stem cells from mice promoted asymmetric cell division while EGF treatment rescued the reduction of asymmetric division in DMD mice model, resulting in enhanced regeneration [17]. Since the role of EGF signaling in tissue is cell dependent and all the studies performed in muscle were using mice samples, we decided to study the activation of EGFR in vitro using isolated FAPs and myoblasts from human samples.

## Materials and methods

### Human skeletal muscle explant culture

Muscle biopsies were collected from healthy controls during surgeries (RVI) and from DMD patients diagnosed at Newcastle Hospital NHS Foundation Trust or Hospital Sant Joan de Déu, Barcelona. FAPs and myoblasts were isolated from muscle samples using anti-PDGFRα-biotin and CD56-FITC antibodies, as previously described [18].

### In vitro differentiation assays

Human primary FAPs were seeded at 5000 cells/cm^2^ in 96-well plates with FAP growth medium (DMEM-GlutaMAX, 20% FBS, 1% PS, 2.5 ng/mL bFGF). Adipogenic differentiation was induced with adipogenic medium (StemPro, Gibco) for 6 days, and fibrogenic differentiation with DMEM-GlutaMAX, 10% FBS, and 5 ng/mL TGFβ for 3 days. Spontaneous differentiation was evaluated using basal media for the same durations. Recombinant EFEMP1 and EGF (R&D Systems) were added from the start of differentiation.

Human immortalized myoblasts were obtained from Institut de Myologie [19] underwent myogenic differentiation with DMEM-GlutaMAX, 5% Horse Serum, and 1% PS for 9 days. The effects of EGF and EFEMP1 (80 ng/mL) on differentiation were assessed by fusion index and myotube area at day 7, using myosin heavy chain staining (clone MF20). Myotube hypotrophy was analyzed by comparing treated and untreated conditions. Gene expression was measured at days 0, 3, 6, and 9.

### Cell staining

Fibrogenic differentiation (collagen-1) and adipogenic differentiation (perilipin-1) were analyzed using quantitative in-cell western. Plates were fixed with 4% PFA, washed, and blocked with casein. Primary antibodies were incubated overnight at 4 °C. Secondary antibodies and CellTag IRDye 700 were applied for 1 h. Fluorescent signals were measured using the Odyssey Imaging system and normalized to cell count.

For immunofluorescence, cells were fixed with 4% PFA and blocked in UltraCruz® Blocking Reagent for 1 h. Primary antibodies against MF20 (myosin heavy chain), alpha-SMA (α-smooth muscle actin), FABP4, and fibronectin-1 were incubated overnight at 4 °C. After washing, appropriate Alexa Fluor-conjugated secondary antibodies were applied for 1 h at room temperature, followed by nuclear staining with DAPI. For lipid staining, Oil Red O (ORO) staining was performed on fixed cells using a working solution of ORO for 15 min, followed by thorough washing in 60% isopropanol and water. Images of random fields were captured using a Zeiss Axioimager and analyzed with ImageJ software.

### ELISA

Supernatant from healthy and DMD-FAPs, as well as serum from healthy and DMD patients, was collected. Serum was separated by centrifuging blood at 1600 × *g* for 9 min at 4 °C, aliquoted, and stored at −80 °C. Fibulin-3 (EFEMP1) and EGF levels were quantified using commercial ELISA kits (Abcam). Samples were measured in duplicate using a Varioskan™ LUX microplate reader (Thermo Fisher).

### Cell viability

The effect of EFEMP1 and EGF on cell viability was assessed with PrestoBlue reagent (Invitrogen) following manufacturer instructions. Fluorescence was measured at 560 nm excitation and 590 nm emission using a Varioskan reader.

### Proliferation assay

Immortalized myoblasts were seeded at 4500 cells/cm^2^ and treated with 80 ng/mL of EFEMP1 or EGF in 2% FBS media. Positive controls used 20% FBS. Proliferation was measured at 24, 48, and 72 h using the CyQUANT Cell Proliferation Assay Kit with a Varioskan reader.

### Migration assay

Migration assays were conducted in 96-well plates with inserts for 72 h. Cells were plated at 1500 cells/cm^2^, treated with 25 µg/mL mitomycin C for 1 h, and then exposed to EGF or EFEMP1 in DMEM with 2% FBS. Migration was tracked using the IncuCyte® system, with images taken every 4 h and analyzed via the Fiji Trackmate package.

### Real-time PCR

Gene expression was assessed using the TaqMan Fast Advanced Cells-to-CT Kit per the manufacturer’s protocol. Cells were lysed, mixed with RT Master Mix, and cDNA synthesized. qPCR was performed with Fast Advanced Master Mix on a QuantStudio™ 7 Flex Real-Time PCR System. Probes included TRIM63, FBXO32, MEF2C, PAX7, MyoD1, MYOG, and GAPDH. Relative quantification was calculated using the comparative Ct method.

### Bioinformatic analysis

Sample analysis utilized various R packages. Seurat (v4.1.0) was employed for sample integration and unsupervised clustering, while CellChat was used for ligand-receptor characterization and cell-cell communication prediction.

### Statistics

The sample size was determined based on the practical limitations of obtaining primary muscle biopsies from both DMD patients and healthy controls. We focused on maximizing biological relevance and experimental reproducibility by ensuring the technical and biological replicates was sufficient to observe consistent trends across downstream assays. We confirmed that data on cell population did not follow normal distribution using the Shapiro–Wilk test and therefore used nonparametric studies, specifically Mann–Whitney U test for independent groups and Wilcoxon rank test to identify significant differences in cell population between samples from snRNAseq dataset. Statistical among the groups were analyzed using one-way ANOVA Test. When ANOVA revealed significant differences, the Tukey post hoc test was performed. A two-way ANOVA followed by Tukey’s post hoc test was used when groups were considered as independent factors. All experiments were performed in triplicate. GraphPad Prism version 10 was used for all statistical tests and plot generation (La Jolla, CA, USA).

## Results

### In-silico analysis of EGFR signaling in muscle tissue

**Fig. 1:**
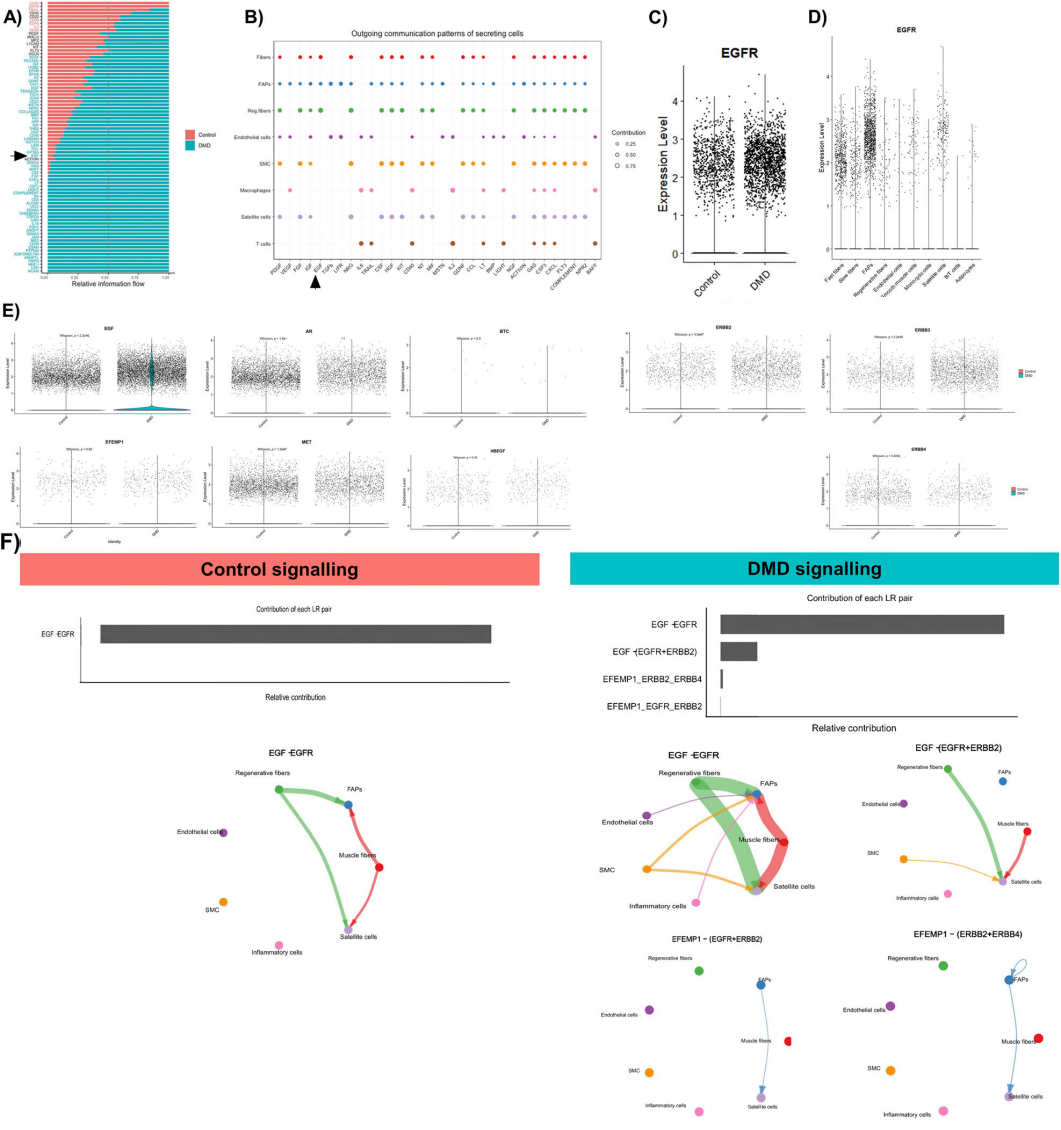
In silico analysis of EGF pathway in muscle. **A** Bar graph showing the weight of each signaling pathway in muscle tissue from healthy control and DMD patients. **B** Outgoing communication pattern of secreting cells showing the involvement of each pathway by the different cells present in the muscle. **C** Violin plot sowing the expression levels of EGFR in muscle tissue from healthy subjects and DMD patients. **D** Violin plot showing the expression levels of EGFR by the different cells present in the muscle tissue. **E** Violin plot showing the expression levels of the different ligands of the EGF pathway and the different ERBB receptors in healthy subjects and DMD patients. **F** Relative contribution of each ligand-receptor pair to the overall communication network of EGF signaling pathway and chord diagram showing the cell-cell communication between the different cells present in the muscle mediated by the ligand-receptor involved in the EGF pathway. SMC smooth muscle cells. Datasets correspond to *n* = 7 DMD patients and *n* = 5 gender marched controls. Data are shown as means ± SD. Results were statistically analyzed using the Wilcoxon test to identify significant differences.

Our previous work on muscle samples from DMD patients and healthy controls showed upregulation of the EGF signaling pathway in DMD [10]. We found that EGF signaling involved communication between various muscle cells, with EGF secreted by FAPs, fibers, and regenerative fibers (Fig. 1A, B). When we analyzed the expression levels of the EGFR at the single nuclei dataset, we observed that it was increased in DMD (Fig. 1C) and that its expression was predominant in FAPS although satellite cells and muscle fibers also expressed the receptor (Fig. 1D). We also observed increased expression of EGF and EGF containing fibulin extracellular matrix protein 1 (EFEMP1) in DMD, while MET and AREG were more prominent in controls. BTC and HBEGF did not show any difference between condition (Fig. 1E). When we analyzed the different receptor subtypes of the EGFR family, we observed that ERBB2 and ERBB3 was increased in DMD condition while ERBB4 was decreased (Fig. 1E).

Using CellChat, we analyzed the intercellular communication and found differences in cell interactions between control and DMD samples. As shown in Fig. 1F, among all known ligand-receptor pairs, control samples were dominated by the EGF-EGFR interaction, while in DMD, we observed a substantial contribution of other ligand and receptors; EGF ligand binding the EGFR-ERBB2 receptor, EFEMP1 ligand binding the EGFR-ERBB2 receptor but also the ERRB-ERBB4 receptor. EGF was secreted by muscle fibers, endothelial cells, and immune cells in DMD, whereas in controls, it was primarily secreted by regenerative fibers and muscle fibers. EFEMP1, released mainly by FAPs, affected FAPs in an autocrine manner acting through the ERBB2-ERBB4 receptor and satellite cells in a paracrine manner through EGFR-ERBB2 and ERBB2-ERBB4. We decided to validate these results in vitro with EGF and EFEMP1.

### Analysis of EGF and EFEMP1 expression in vitro

**Fig. 2:**
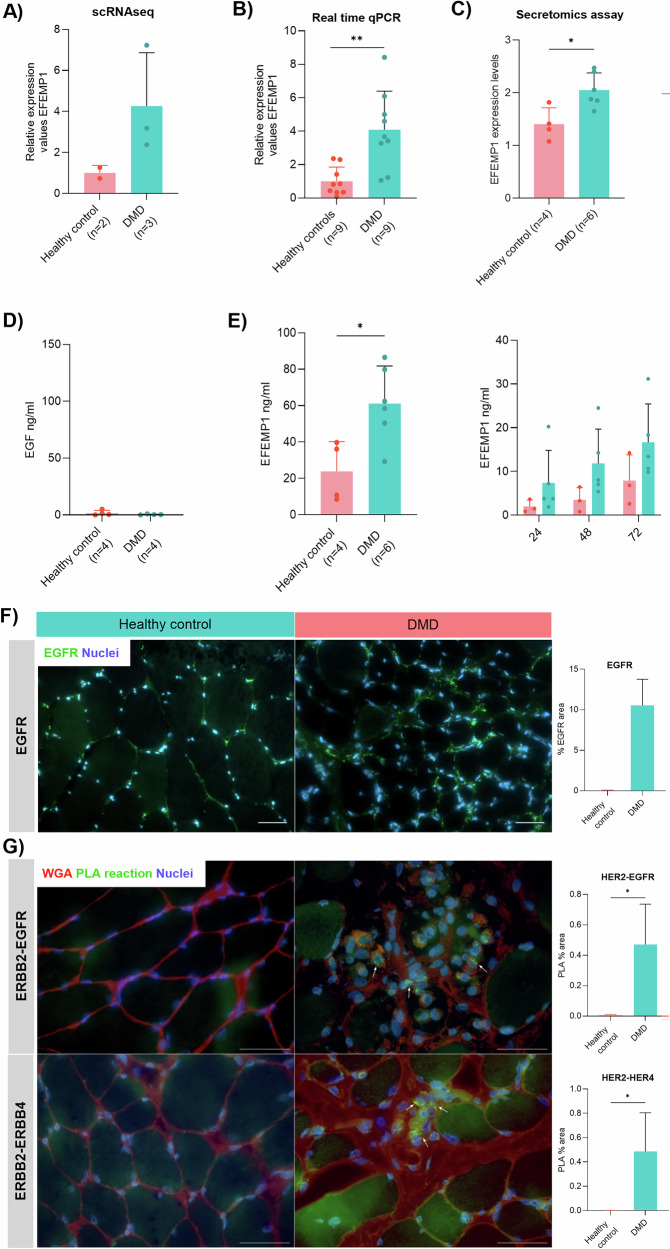
In vitro validation of EGF and EFEMP1 expression in FAPs. **A** Bar graph showing the relative expression values of EFEMP1 in the dataset obtained from single-cell RNA sequencing. Data points correspond to individual biological samples, with healthy controls (*n* = 2) and DMD patients (*n* = 3). **B** Bar graph showing the relative expression values of EFEMP1 in real-time qPCR analysis. Data points correspond to individual biological samples, with healthy controls (*n* = 9) and DMD patients (*n* = 9). **C** Bar graph showing the relative expression values of EFEMP1 in the dataset obtained from the proteome analysis of supernatant. Data points correspond to individual biological samples, with healthy controls (*n* = 4) and DMD patients (*n* = 6). **D** Bar graph showing the concentration of EGF analyzed by ELISA. Data points correspond to individual biological samples, with healthy controls (*n* = 4) and DMD patients (*n* = 4). **E** Bar graphs showing the concentration of EFEMP1 released from FAPs and the concentration release over 3 days. Data points correspond to individual biological samples, with healthy controls (*n* = 4) and DMD patients (*n* = 6). **F** Representative images of muscle tissue showing the expression of EGFR and nuclei. Scale bar = 250 µM. And a bar graph representing the % of EGFR staining per field. An average of *n* = 3 independent replicates is shown. **G** Detection of EGFR-ERBB2 and ERBB2-ERBB4 dimerization using Duolink PLA in muscle tissue. White arrows show the PLA reaction. Scale bar = 500 µM. Five random images were acquired using Hoesch (blue nuclei) and WGA staining (red signal). The bar graph shows the % area of positive PLA reaction. An average of *n* = 3 independent replicates is shown. Data are shown as means ± SD. Results were statistically analyzed using Mann–Whitney U test to identify significant differences. Statistical significance was set at *P < 0.05 and **P < 0.01.

We confirmed the expression of EFEMP1 and EGF in FAPs. Single-cell RNA sequencing and qPCR showed increased EFEMP1 expression in DMD FAPs (Fig. 2A, B) [20] while secretomic analysis of the supernatant of cultured FAPs and ELISA analysis further confirmed these findings (Fig. 2C). We observed increased release of EFEMP1 in the supernatant of DMD FAPs while EGF was not even detected (Fig. 2D, E). Additionally, we observed that EFEMP1 concentration progressively increased in the supernatant in a time-dependent manner being the secretion always higher in the DMD FAPs culture (Fig. 2E).

We also validated the results from the snRNAseq ex vivo using muscle tissue from healthy control and DMD patient. We confirmed that the EGFR is increased in DMD tissue (Fig. 2F) and also that the heterodimerization of EGFR-ERBB2 and ERBB-ERBB4 is mainly present in the DMD condition (Fig. 2G).

### Activation of EGFR and ErbB/HER receptors in FAPs

**Fig. 3:**
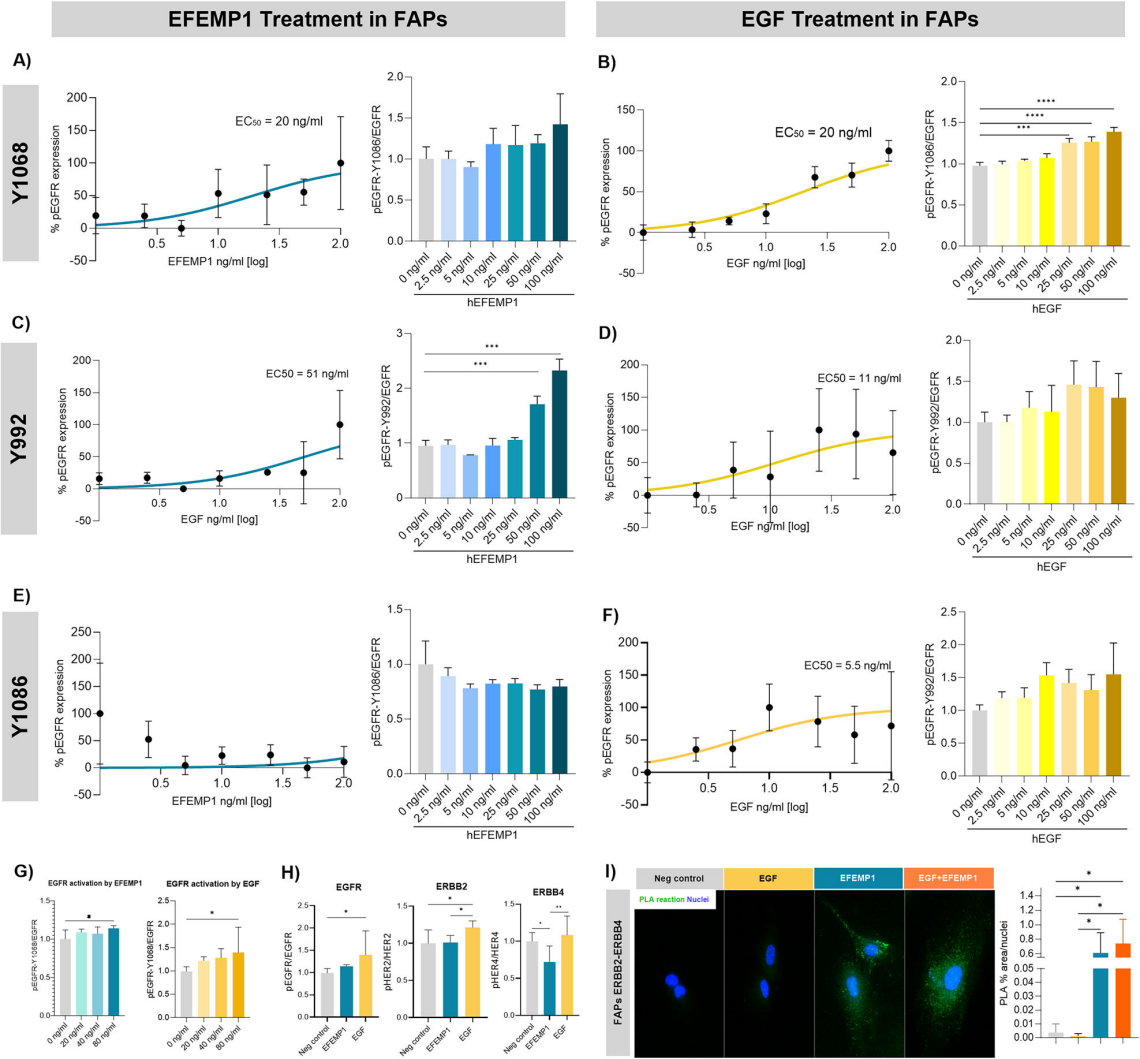
Activation of EGFR in human FAPs using EFEMP1 and EGF. **A** Dose-response effect of EFEMP1 on EGFR site phosphorylation of Tyr1068. An average of *n* = 3 independent replicates is shown. **B** Dose-response effect of EGF on EGFR site phosphorylation of Tyr1068. An average of *n* = 3 independent replicates is shown. **C** Dose-response effect of EFEMP1 on EGFR site phosphorylation of Tyr992. **D** Dose-response effect of EGF on EGFR site phosphorylation of Tyr992. An average of *n* = 3 independent replicates is shown. **E** Dose-response effect of EFEMP1 on EGFR site phosphorylation of Tyr1086. An average of *n* = 3 independent replicates is shown. **F** Dose-response effect of EGF on EGFR site phosphorylation of Tyr1086. An average of *n* = 3 independent replicates is shown. **G** Bar graph showing the activation of EGFR-Tyr1068 by EFEMP1 and EGF at 20, 40, and 80 ng/ml. An average of *n* = 3 independent replicates is shown. **H** Bar graph showing the activation of ERBB2, ERBB3, and ERBB4 by EFEMP1 and EGF at 80 ng/ml. Phosphorylation was normalized to receptor levels, and the data were further normalized to the negative control condition in each experiment. An average of *n* = 3 independent replicates is shown. **I** Detection of ERBB2-ERBB4 dimerization using Duolink PLA in FAPs after EGF and EFEMP1 activation in vitro. The bar graph shows the % area of positive PLA reaction measured against the total nuclei per field. An average of *n* = 3 independent replicates is shown. Data are shown as means ± SD. Results were statistically analyzed using one-way ANOVA followed by the Tukey post hoc test. Statistical significance was set at *P* < 0.05. **P < 0.01; ***P < 0.001.

We examined EGFR signaling by testing phosphorylation of various EGFR residues after treatment with EGF and EFEMP1. Phosphorylation of tyrosine residue 1068 (Tyr1068) was detected after both EGF and EFEMP1 treatment at the same IC50 (Fig. 3A, B). Activation of the EGFR-Tyr1068 was confirmed using three doses ranging from the IC50 value detected, being 80 nM the dose that significantly increased the activation of the receptor using EGF and EFEMP1 (Fig. 3G). However, when we compared that activation between factors, the effect of EGF was more potent than EFEMP1 (Fig. 3G). Different IC50 was detected when analyzing residue Tyr992, being EFEMP1 more potent than EGF (Fig. 3C, D). When the Tyr1086 residue was analyzed, EFEMP1 did not show any activation while EGF did it (Fig. 3E, F).

We also investigated ErbB/HER receptor activation and heterodimerization. EGF significantly activated ErbB2/HER2, while EFEMP1 had no effect. For ErbB4, EGF increased activation, whereas EFEMP1 decreased it, highlighting distinct receptor activation by these ligands (Fig. 3H). We observed that according to the snRNAsesq data, EFEMP1 promoted the heterodimerization of ERBB2-ERBB4 and that when both the EGF and EFEMP1 where in the medium these were not competing but still promoting the heterodimers (Fig. 3I).

### In vitro effect of EGF and EFEMP1 on FAPs

**Fig. 4:**
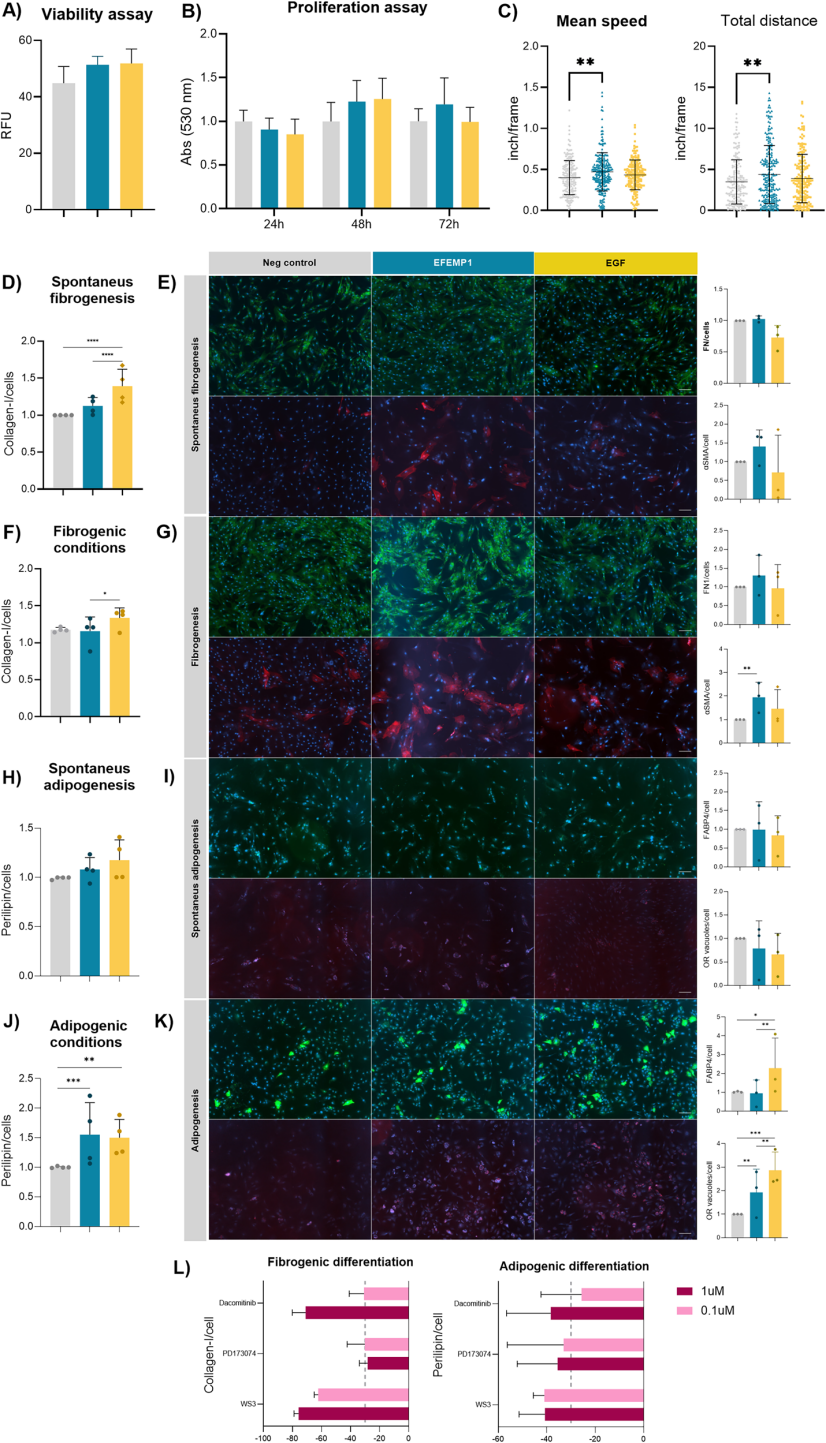
In vitro effect of EFEMP1 and EGF on FAPs. **A** Bar plot representing the viability of healthy human FAPs after treatment with EFEMP1 and EGF. An average of *n* = 3 independent replicates is shown. Results were statistically analyzed using one-way ANOVA, followed by the Tukey post hoc test. **B** Bar plot showing the proliferation data after 24, 48, and 72 h. An average of *n* = 3 independent replicates is shown. **C** Dot plot showing the mean speed and total distance of FAPs after 72 h in culture. Each dot corresponds to each cell analyzed. An average of *n* = 3 independent replicates was used. Results were statistically analyzed using one-way ANOVA, followed by the Tukey post hoc test. **D** Bar graph showing the increase of collagen-I in healthy human FAPs under maintenance media (10% FBS in DMEM) after EGF and EFEMP1 treatment. Data points correspond to individual biological samples (*n* = 4). **E** Representative images of fibronectin-1 and αSMA staining under maintenance media and after EGF and EFEMP1 treatment with bar graphs representing the expression of FN1 and αSMA measured against the total nuclei per field. Data points correspond to individual biological samples (*n* = 3). **F** Bar graph showing the increase of collagen-I in human under fibrogenic conditions after EGF and EFEMP1 treatment. Data points correspond to individual biological samples (*n* = 4). **G** Representative images of fibronectin-1 and αSMA staining under fibrogenic media and after EGF and EFEMP1 treatment with bar graphs representing the expression of FN1 and αSMA measured against the total nuclei per field or inducing fibrogenic conditions with and without EFEMP1 or EGF. Data points correspond to individual biological samples (*n* = 3). **H** Bar graph showing the increase of perilipin in human FAPs after maintenance media with and without EFEMP1 or EGF. Data points correspond to individual biological samples (*n* = 4). **I** Representative images of FABP4 and ORO staining under maintenance media and after EGF and EFEMP1 treatment with bar graphs representing the expression of FABP4 and ORO measured against the total nuclei per field. Data points correspond to individual biological samples (*n* = 3). **J** Bar graph showing the increase of perilipin in healthy human FAPs after adipogenic media with and without EFEMP1 or EGF. Data points correspond to individual biological samples (*n* = 4). **K** Representative images of FABP4 and ORO staining under adipogenic media and after EGF and EFEMP1 treatment with bar graphs representing the expression of FABP4 and ORO measured against the total nuclei per field. Data points correspond to individual biological samples (*n* = 3). Scale bar = 250 µM. **L** Bar graph showing the percentage of inhibition of fibrogenic and adipogenic differentiation after treating FAPs with 0.1 or 1 µM of three different inhibitors (Dacomitinib, PD173074, and WS3). The gray dotted line represents 30% inhibition. An average of *n* = 3 independent replicates is shown. Data are shown as means ± SD. Results were statistically analyzed using two-way ANOVA, followed by the Tukey post hoc test. Statistical significance was set at *P* < 0.05. **P < 0.01; ***P < 0.001.

We tested the effects of EGF and EFEMP1 on primary FAPs from healthy muscle. Both ligands were non-toxic at the tested dose neither for the cell (Fig. 4A) nor at mitochondrial level (Supplementary Fig. 1A). While EGF and EFEMP1 did not significantly affect FAP proliferation (Fig. 4B), EFEMP1 significantly increased FAP migration speed and distance, whereas EGF had no effect (Fig. 4C). Other analysis, such as the directional change rate or the linearity of forward progression had no differences when either EGF or EFEMP1 were in the media (Supplementary Fig. 1B).

We also analyzed the differentiation potential of FAPs. EGF increased the spontaneous fibrogenesis by upregulating Collagen-I (Fig. 4D). However, EFEMP1 promoted an increase of alpha-smooth actin (αSMA) under fibrogenic conditions (Fig. 4E, G). Neither ligand induced spontaneous adipogenesis, but both increased perilipin expression and the number of lipidic vacuoles under adipogenic media (Fig. 4J, K). However, the effect of EGF on adipogenesis was more evident by promoting a higher expression of FABP4 (Fig. 4K).

To validate the role of EGFR signaling in differentiation, we used EGFR inhibitors (WS3, Dacazomib, PD1070), which reduced both fibrogenic and adipogenic differentiation at high and low doses (Fig. 4L). These findings suggest that EGFR signaling plays a key role in FAP differentiation and that anti-EGFR inhibitors could help reduce fibro-fatty tissue expansion in patients.

### In vitro effect of EGF and EFEMP1 on myoblasts

**Fig. 5:**
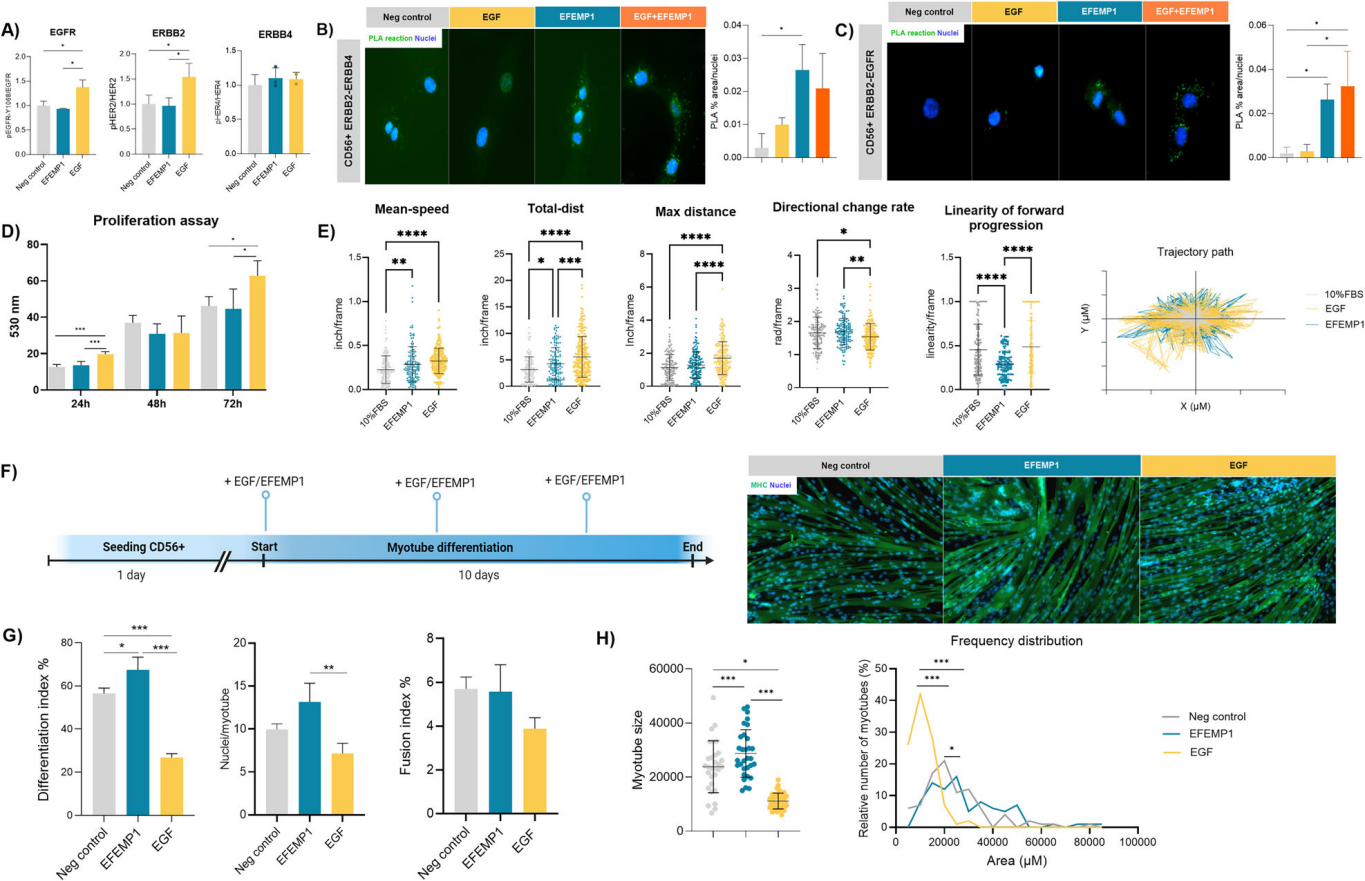
In vitro effect of EFEMP1 or EGF on myoblasts. **A** Bar graph showing the activation of EGFR, ERBB2, and ERBB4 by EFEMP1 and EGF at 80 ng/ml. Phosphorylation was normalized to receptor levels and the data were further normalized to the negative control condition in each experiment. An average of *n* = 3 independent replicates is shown. **B** Detection of ERBB2-ERBB4 dimerization using Duolink PLA in myoblasts after EGF and EFEMP1 activation in vitro. Bar graph shows the % area of positive PLA reaction measured against the total nuclei per field. An average of *n* = 3 independent replicates is shown. **C** Detection of ERBB2-EGFR dimerization using Duolink PLA in myoblasts after EGF and EFEMP1 activation in vitro. Bar graph shows the % area of positive PLA reaction measured against the total nuclei per field. An average of *n* = 3 independent replicates is shown. **D** Bar plot showing the proliferation data after 24, 48, and 72 h. An average of *n* = 3 independent replicates is shown. Results were statistically analyzed using two-way ANOVA, followed by the Tukey post hoc test. **E** Dot plot showing the mean speed, total distance, maximum distance, directional change rate of myoblasts, linearity of forward progression and a trajectory path and after 72 h in culture. Each dot corresponds to each cell analyzed. An average of *n* = 3 independent replicates was used. **F** Timeline scheme of the differentiation of myoblasts with EFEMP1 or EGF treatment and representative images of myotubes after differentiation with or without EFEMP1 and EGF. **G** Bar plot showing the differentiation, fusion index and ratio of nuclei per myotube of untreated and treated myoblasts with EFEMP1 or EGF after the differentiation process. An average of *n* = 3 independent replicates is shown. **H** Myotube fiber size graph and frequency of distribution graph showing differences between non-treated and EFEMP1 or EGF-treated myoblasts after differentiation. Each dot corresponds to each myotube analyzed. An average of *n* = 3 independent replicates was used. Data are shown as means ± SD. Results were statistically analyzed using one-way ANOVA, followed by Tukey post hoc test. Statistical significance was set at *P* < 0.05. **P < 0.01; ***P < 0.001.

We investigated the effects of EGF and EFEMP1 on myoblasts and myotubes using an immortalized cell line from a healthy individual. EGF activated EGFR and ERBB2, while EFEMP1 did not activate EGFR or ERBB4 (Fig. 5A). According to the snRNAseq dataset, EFEMP1 promoted the heterodimerization of ERBB2-ERBB4 and ERBB2-EGFR in myoblasts, something that was not suppressed when both ligands were together in the media (Fig. 5B, C). EGF significantly increased myoblast proliferation at 24 and 72 h, but EFEMP1 had no effect (Fig. 5D). The transient decrease at 48 h may reflect a temporary internalization of EGFR. Both ligands enhanced migration, with EFEMP1 reducing the linearity of movement, while EGF maintained linearity and decreased directional changes (Fig. 5E).

In myotube differentiation conditions, EFEMP1 promoted myoblast differentiation and increased myotube size, while EGF reduced differentiation and led to smaller myotubes (Fig. 5F–H). When factors were added once myotubes were already differentiated (at day 7) neither EGF nor EFEMP1 affected the differentiation or fusion index of mature myotubes (Supplementary Fig. 1C). However, EGF also led to smaller myotubes (Supplementary Fig. 1D).

### Transcriptional changes and muscle contractility effects of EGF and EFEMP1 in myotube differentiation

**Fig. 6:**
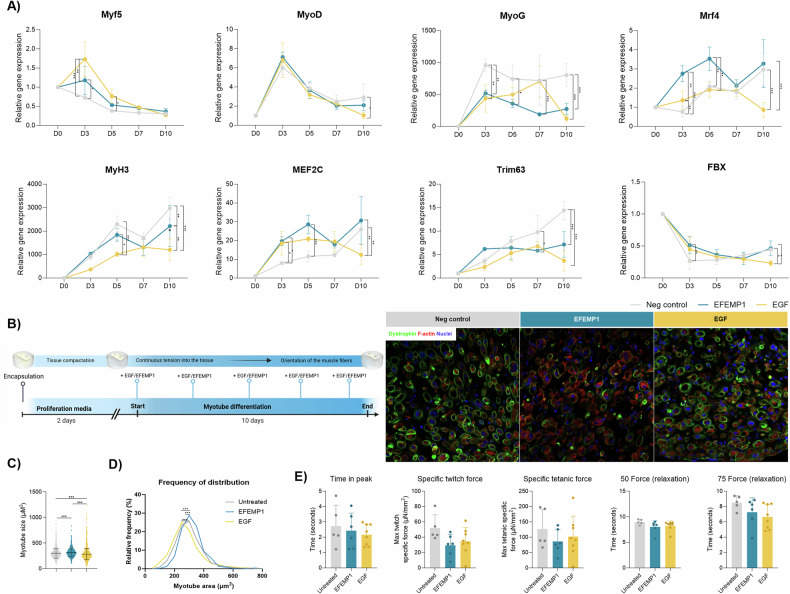
Effect of EFEMP1 and EGF on myotube differentiation myogenic genes and in 3D culture. **A** Relative expression of Myf5, MyoD, MyoG, Mrf4, MyH3, Mef2c, Trim63, and Fbx analyzed after differentiation process in non-treated and EFEMP1 or EGF-treated myoblasts. Results were statistically analyzed using two-way ANOVA, followed by the Tukey post hoc test. An average of *n* = 3 independent replicates is shown. **B** Timeline scheme of the differentiation of myoblasts with EFEMP1 or EGF treatment and representative images of 3D culture after differentiation with or without EFEMP1 and EGF. **C** Frequency of distribution graph showing differences between non-treated and EFEMP1 or EGF-treated myoblasts after differentiation. An average of *n* = 3 independent replicates is shown. **D** Myotube fiber size graph showing differences between non-treated and EFEMP1 or EGF-treated myoblasts after differentiation. An average of *n* = 3 independent replicates is shown. **E** Bar graph showing contractile dynamics of the 3D muscle tissue after 10 days of differentiation. Time in peak (seconds), specific twitch force (µN/mm^2^), specific tetanic force (µN/mm^2^), time to relaxation to the 75% and 50% of the maximum force performed by tissues were measured. Each dot corresponds to each 3D myotube analyzed. An average of *n* = 3 independent replicates was used. Results were statistically analyzed using one-way ANOVA, followed by the Tukey post hoc test. Data are shown as means ± SD; Statistical significance was set at *P* < 0.05, **P < 0.01, ***P < 0.001.

To study EGF role in myogenesis, we assessed the expression of myogenic transcription factors during myotube differentiation with EGF or EFEMP1. Both ligands increased Myf5 at day 3, while only EGF increased it at day 5. EFEMP1 reduced MyoG throughout, while EGF only reduced it on days 3 and 10. EGF also reduced MyoD expression at the end of differentiation. EFEMP1 consistently upregulated Mrf4 and MEF2C, while EGF only increased them at day 3. Myosin heavy chain expression decreased with EGF at day 10, and pro-atrophic factors Trim63 and FBX showed differing patterns of regulation (Fig. 6A).

We further examined the effects in a 3D muscle model, where contraction was analyzed after 10 days of differentiation. EGF-treated myotubes were smaller in size but more numerous, while EFEMP1-treated myotubes were larger (Fig. 6B–D). Force generation showed a non-significant change in all parameters, for both EGF and EFEMP1-treated muscles compared to controls (Fig. 6E and Supplementary Fig. 1E).

## Discussion

This study explores the role of EGF signaling in DMD, showing that EGF signaling is increased in DMD muscle samples compared to healthy controls. We observed different ligands and receptors activated in DMD muscles, suggesting that EGF signaling behaves differently in pathological conditions. Specifically, EGF and EFEMP1, two key ligands, modulate muscle cell behavior in distinct ways.

The epidermal growth factor (EGF) is known to modulate a variety of cellular functions, including cell proliferation, differentiation, and migration [21, 22]. EGF signaling has been involved in a myriad of cellular processed such as migration of cancer cells and the invasion of tissues, development of myelin, and differentiation of oligodendrocytes [23, 24]. On the other hand, the epidermal growth factor-containing fibulin-like extracellular matrix protein 1 (EFEMP1), also called fibulin-3, is a member of the fibulin family of extracellular glycoproteins [25]. Fibulins are also involved in several cell processes such as modulation of cell morphology, growth adhesion, and motility [26, 27]. Although the role of EFEMP1 has been previously described in cancer, its effect in muscle has never been addressed [28].

Based in our observation of existing different EGF intercellular communication between healthy and DMD muscles in the in silico analysis, we decided to study the in vitro response of FAPs and myoblasts treated with EGF and EFEMP1. We observed that EGF significantly enhanced FAPs fibrogenic differentiation, aligning with previous studies that have characterized EGF signaling in the expansion of the ECM in other tissues and diseases, such as cancer or pulmonary fibrosis [29]. However, EFEMP1 promoted an increase in αSMA expression—an important factor for cellular structural rearrangement—even in the absence of fibrogenic differentiation and enhanced the migratory capacity of FAPs. These mechanisms suggest a role for EFEMP1 in the initial activation of FAPs. This is consistent with previous findings in osteosarcoma cells that highlight EFEMP1’s role in enhancing migratory and invasive capabilities [30]. Additionally, we observed that both EGF and EFEMP1 increased the adipogenic differentiation potential of FAPs when cells were grown under adipogenic conditions, being EGF more potent than EFEMP1.

FAPs play a critical role in muscle regeneration following injury but can contribute to fibrosis and fat replacement when overactivated, as seen in DMD [31–34]. Our findings suggest that dysregulated EGFR signaling in DMD leads to increased FAP migration and differentiation, potentially exacerbating fibrosis and fat deposition. In vitro, EGFR inhibitors reduced these processes, highlighting the therapeutic potential of targeting EGFR signaling in DMD.

The different roles of both factors were also observed in myoblasts. EGF treatment resulted in increased proliferation and migration but also led to smaller myotubes, suggesting that EGF promotes early-stage myoblast proliferation but limits later differentiation. The increase in Myf5 expression, which is associated with cell proliferation and reduced differentiation potential [17], may explain this effect. Additionally, EGF treatment reduced the expression of other myogenic factors like MyoD, MEF2C, Mrf4, and myogenin, leading to smaller myotubes as confirmed by lower Myh3 expression at the end of differentiation, which was consistent with smaller myotubes when EGF was added at day 7 of differentiation. While EGF has been shown to enhance satellite cell proliferation in healthy muscle [35], the persistent activation in DMD may limit satellite cell differentiation, potentially leading to muscle dysfunction. Additionally, research done in skeletal muscles of patients with COPD suggest that increased EGF leads to reduction of the number of type I slow muscle fibers and increase in type II fast fibers [16]. If that is the case, this could have a negative impact on DMD patients, as it is well known that fast fibers are more susceptible to contraction-induced damage [36], further amplifying the negative role of EGF in skeletal muscle homeostasis in DMD patients.

In contrast, EFEMP1 did not significantly affect myoblast proliferation but enhanced both their migration and differentiation potential. EFEMP1 treatment increased migration but myoblasts lost their linearity when moving, which could translate into an increased ability of the cells to move from one fiber to another and participate in the muscle regeneration. The linearity of myoblasts migration is important during regeneration after an acute injury, since myoblasts are activated and follow a directional migration towards the situ of injury above the basal laminal [37]. The wider myotubes observed could be due to increased expression of myogenic factors like Mrf4 and MEF2C, which promote differentiation. Additionally, MEF2C is known to induce hypertrophy in adult muscle fibers, which may explain the larger myotubes observed in our study. These findings are consistent with observations in DMD muscle biopsies, where hypertrophic fibers coexist with smaller, regenerative fibers [10, 38]. We hypothesize that EFEMP1 could increase the size of muscle fibers by promoting satellite cell differentiation, although this did not translate into improved muscle force or functionality in our 3D model.

Our study suggests that both EGF and EFEMP1 appear to play distinct roles in muscle regeneration and pathology. EGF promotes a regenerative response by increasing the number of small fibers, while EFEMP1 may regulate myofiber hypertrophy. However, neither factor improved muscle force or functionality in our models, suggesting that while these factors influence muscle behavior, they do not enhance muscle performance in DMD.

**Fig. 7:**
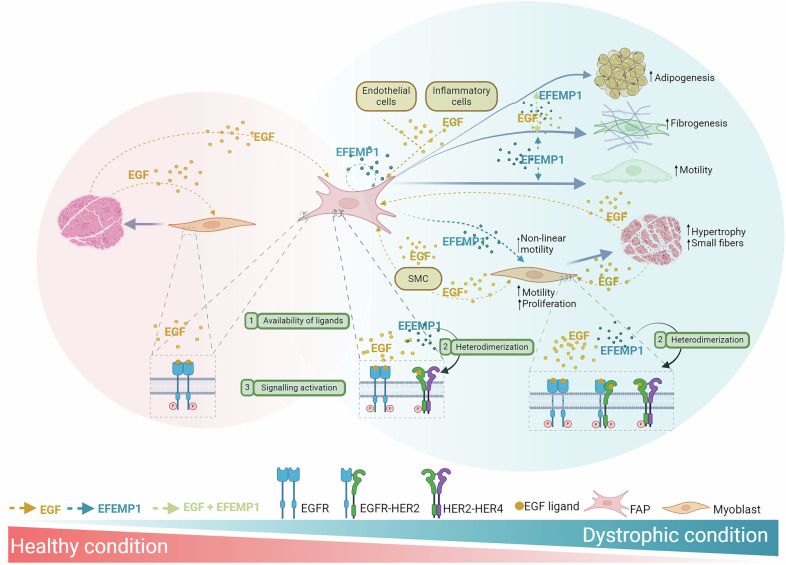
Representative scheme of EGFR signaling and regulation in muscle. Activation of EGF signaling occurs through paracrine and autocrine mechanisms. In healthy condition, EGFR is activated by EGF both in FAPs and myoblasts promoting the homeostasis of the muscle. However, in dystrophic condition the signaling is increased by higher release of EGF by other cells and also the release of EFEMP1 by FAPs (1). The presence of EFEMP1 in the muscle promotes the heterodimerization (2) of HER2-HER4 in both myoblasts and FAPs and the heterodimerization of EGFR-HER2 in myoblasts. That heterodimerization increases the signal by EGF promoting a higher response of the EGF-signaling pathways. Furthermore, the activation of EGF and EFEMP1 through EGFR phosphorylates different residues od the receptor, creating a different response in the cell (3). While in healthy condition there is a maintenance of regeneration of the muscle leaded by the cross-talk of different cells present in the tissue, in dystrophic condition the presence of EGF and EFEMP1 promotes an increased adipogenesis, fibrogenesis and activation of FAPs by an increased migration. In myogenesis, EGF and EFEMP1 promotes an increased hypertrophy of the fibers together with small fibers, which lead to lower regeneration and a disrupted architecture of the muscle.

The differential effects of EGF and EFEMP1 on FAPs and myoblasts may be explained by several molecular mechanisms:

(1) The differential secretome of DMD FAPs affecting the availability of ligands in each condition has the potential to initiate distinct signaling pathways resulting in different response. In healthy muscle, EGF is released by regenerating fibers and acts on FAPs and satellite cells. In contrast, DMD muscle shows increased EGF release from various cell types, including inflammatory, smooth muscle, and endothelial cells. Our in vitro studies confirmed that EGF activates EGFR in FAPs and both EGFR and ErbB2 in myoblasts. Additionally, EFEMP1 release is increased in DMD FAPs and acts through EGFR and ErbB2, ErbB4 receptors, influencing the behavior of both FAPs and satellite cells (Fig. 7).

(2) The different receptor activation patterns may also explain the distinct effects of EGF and EFEMP1. We demonstrated that ErbB2–ErbB4 and ErbB2–EGFR heterodimerization were significantly increased in DMD tissue, and that EFEMP1 promoted this heterodimerization, potentially amplifying the response to EGF. This process has been described in cancer-associated fibroblasts, where receptor heterodimerization modulates cellular responses [39]. In our study, EFEMP1 treatment enhanced FAP migration, possibly by promoting heterodimerization through ErbB2, which is known to regulate actin polymerization and cytoskeletal reorganization necessary for cell migration [40].

(3) The different ligands can lead to different molecular mechanisms. We found that EGF and EFEMP1 activate different signaling pathways through distinct patterns of phosphorylation in EGFR residues. This suggests that the ligands initiate distinct molecular responses, potentially influencing cell behavior in different ways. While EGF promotes an activating signaling response, EFEMP1 seems to maintain EGFR and ErbB receptors in an inactive state, probably acting as a scaffold protein, which may enhance the effect of EGF by allowing it to activate EGFR-ErbB2 signaling more effectively.

In conclusion, while other mechanisms likely contribute to DMD pathogenesis, our study underscores the importance of EGFR signaling in disrupting muscle cell communication. We demonstrated for the first time that heterodimers of ErbB-EGFR family are significantly increased in DMD and that EFMEP1 promoted this heterodimerization. The EGFR signaling pathway plays a critical role in DMD by modulating FAP and myoblast behavior contributing to fibrotic and adipogenic tissue expansion, as well as impaired muscle regeneration. Targeting EGFR signaling may help reduce fibrofatty differentiation in DMD, but the complexity of this pathway requires further investigation. Understanding how different ligands influence EGFR signaling and receptor heterodimerization will be essential for developing new therapeutic options for muscular dystrophies.

## Supplementary information

Supplementary material

Supplementary Figure 1

Reagents

## Data Availability

All data will be available in an open repository upon acceptance of the manuscript.
