# Correction: Investigating the role of EGFR signalling in muscle dystrophies: implications for Duchenne muscular dystrophy

**DOI:** 10.1038/s41419-026-08544-0

**Published:** 2026-04-17

**Authors:** Esther Fernández-Simón, Ainoa Tejedera-Villafranca, Xiomara Fernández-Garabay, James Clark, Alexandra Monceau, Elisa Villalobos, Dan Cox, Javier Ramón Azcón, Juan M. Fernández-Costa, Jordi Diaz-Manera

**Affiliations:** 1 John Walton Muscular Dystrophy Research Centre, Institute of Genetic Medicine, Newcastle University, Newcastle upon Tyne, UK; 2 Institute for Bioengineering of Catalonia (IBEC), The Barcelona Institute of Science and Technology (BIST), Barcelona, Spain; 3 Institució Catalana de Recerca i Estudis Avançats (ICREA), Barcelona, Spain

**Keywords:** Molecular biology, Pathogenesis

Correction to: *Cell Death & Disease* 10.1038/s41419-025-08193-9, published online 09 January 2026

In this article, the author name “Jordi Diaz-Manera” was incorrectly written as “Jordi Diaz Manera”.

The original article has been corrected.

