# Supplementary material for: Investigating the role of EGFR signalling in muscle dystrophies: implications for Duchenne muscular dystrophy
Source: Cell Death Dis. 2026 Jan 9;17(1):18. doi: 10.1038/s41419-025-08193-9 (PMC12789545; doi:10.1038/s41419-025-08193-9)
Supplement: Supplementary file 1 — Supplementary material [file 41419_2025_8193_MOESM1_ESM.docx]

**MATERIAL & METHODS**

**Tissue staining**

Frozen muscle tissue sections (7 μm) were air-dried, fixed in 4% PFA for 10 minutes, and permeabilized with 0.1% Triton X-100 for 10 minutes at room temperature. After blocking with UltraCruz® Blocking Reagent solution for 1 hour, sections were incubated overnight at 4°C with a primary antibody against EGFR. Following washes in PBS, sections were incubated with an Alexa Fluor-conjugated secondary antibody for 1 hour at room temperature. Nuclei were counterstained with DAPI, and sections were mounted using antifade mounting medium. using ImageJ.

**Mitochondrial function**

The effect of EFEMP1 and EGF on mitochondrial function was assessed in healthy human FAPs with Tetramethylrhodamine methyl ester (TMRM) and MitoSox assay following manufacturer instructions. Fluorescence was measured using a Varioskan reader.

**Proximity Ligation Assay (PLA)**

Proximity ligation assays were performed using the Duolink® In Situ Green Kit (Sigma-Aldrich) following the manufacturer’s protocol. FAPs and myoblasts were seeded on chambered slides and treated with or without EGF (80 ng/mL) or EFEMP1 (80 ng/mL) or both of them for 10 minutes. Cells were fixed in 4% paraformaldehyde, washed with PBS, and blocked in Duolink blocking buffer. Primary antibody pairs targeting EGFR–ErbB2 or ErbB4–ErbB2 were incubated overnight at 4°C. After washing, PLA probes were applied, followed by ligation and amplification steps. Nuclei were counterstained with DAPI, and the PLA signals (green signal) were visualized using a Zeiss Axioimager. Quantification was performed by counting the positive PLA signal per cell using ImageJ.

When muscle tissue was used, PLA was performed on muscle sections of 7 µM and following the same protocol. At the time of adding DAPI, WGA was also added to visualize the muscle structure.

**In Vitro Functional 3D Muscle Differentiation Assays**

Human 3D skeletal muscle tissues were created using polydimethylsiloxane (PDMS) molds. After cleaning and treating molds with 2% Pluronic® F-127 solution in PBS overnight, human immortalized myoblasts were encapsulated at 2.5 × 10⁷ cells/mL in a hydrogel mixture. The hydrogel included 30% Matrigel®, 2 U/mL thrombin, and 4 mg/mL fibrinogen, polymerized at 37°C for 30 minutes. Tissues were cultured in growth medium (skeletal muscle basal medium with supplements, 10% FBS, penicillin-streptomycin, and 1 mg/mL 6-aminocaproic acid).

After two days, tissues were switched to differentiation medium containing EFEMP1 or EGF (80 ng/mL) for 10 days. Differentiation medium included DMEM high glucose with supplements (KnockOut™ Serum Replacement, Insulin-Transferrin-Selenium-Ethanolamine, Penicillin-Streptomycin-Glutamine, and ACA). Half of the medium was replaced every two days.

**Electrical Pulse Stimulation (EPS) and Force Measurement**
After 12 days of differentiation, muscle tissues were electrically stimulated to induce contraction. PDMS molds were placed in a 24-well plate with fresh differentiation medium, set on a Zeiss Axio Observer Z1/7 microscope with an incubator. A custom device with graphite electrodes and a pulse generator applied square-wave pulses (1 V/mm, 1 ms, 1–50 Hz). Brightfield videos recorded pillar movement during contraction to measure contractile forces, normalized by tissue cross-sectional area. Videos were processed as previously described.

**Cryosectioning, immunohistochemistry, imaging and image analysis of 3D skeletal muscle tissues**

3D skeletal muscle tissues were fixed in 10% formalin for 30 minutes at room temperature, washed with PBS, and incubated in 30% sucrose solution at 4°C for 48 hours. Samples were embedded in OCT compound using chilled isopentane and sectioned at 20 μm thickness with a cryostat (Leica CM1900). Sections were placed on SuperFrost Plus™ Adhesion slides.

Tissue sections were encircled with a PAP pen, permeabilized with PBS-T (0.1% Triton-X in PBS) for 10 minutes, and blocked with UltraCruz® Blocking Reagent for 30 minutes. Samples were incubated overnight at 4°C with primary antibodies (anti-α-Actinin SAA (1:200) or anti-dystrophin (MANDYS01 and MANDYS106, 1:50 each)) in blocking buffer. After PBS-T washes, sections were incubated with Alexa Fluor™ 488-conjugated secondary antibody (1:200) and Alexa Fluor™ 594 Phalloidin (1:400) for 45 minutes at room temperature. Sections were mounted with VECTASHIELD Plus Mounting Medium with DAPI, and edges were sealed with transparent enamel.

Fluorescence imaging was performed using a ZEISS LSM800 confocal microscope. Fiji software was used for image analysis. Myotube diameter and area were measured by segmenting the SAA signal using Cellpose 2 and the LabelsToRois ImageJ plugin.

**FIGURE LEGENDS**

**Suplemental figure 1. (A)** Mitochondrial functional assays using Mitosox and TMRM assays in healthy human FAPs after treatment with EFEMP1 and EGF. An average of n=3 independent replicates is shown. Results were statistically analyzed using one-way ANOVA, followed by Tukey post hoc test. **(B)** Dot plot showing the linearity of forward progression and the directional change rate of FAPs after 72 hours in culture. Each dot corresponds to each cell analysed. An average of n=3 independent replicates was used. Results were statistically analyzed using one-way ANOVA, followed by Tukey post hoc test. **(C)** Timeline scheme of the myoblasts differentiation with EFEMP1 or EGF added at day 7 of differentiation treatment and bar plots showing the differentiation, fusion index and ratio of nuclei per myotube of untreated and treated myoblasts with EFEMP1 or EGF after differentiation process. An average of n=3 independent replicates is shown. Results were statistically analyzed using one-way ANOVA, followed by Tukey post hoc test. **(D)** Frequency of distribution graph and myotube fiber size graph showing differences between non-treated and EFEMP1 or EGF treated at the end of differentiation. An average of n=3 independent replicates is shown. **(E)** Bar graph showing contractile dynamics of the 3D muscle tissue after 10 days of differentiation. Twitch and tetanic displacement (µm), tetanic/twitch index, time to peak (s), tome to contraction to the 50% and 75% of the maximum force performed by tissues was measured. Each dot corresponds to each 3D myotube analysed. An average of n=3 independent replicates was used. Results were statistically analyzed using one-way ANOVA, followed by Tukey post hoc test. Data are shown as means ± SD; Statistical significance was set at P < 0.05, **P < 0.01, ***P< 0.001.
