# Supplementary figures and images for: Investigating the role of EGFR signalling in muscle dystrophies: implications for Duchenne muscular dystrophy
Source: Cell Death Dis. 2026 Jan 9;17(1):18. doi: 10.1038/s41419-025-08193-9 (PMC12789545; doi:10.1038/s41419-025-08193-9)

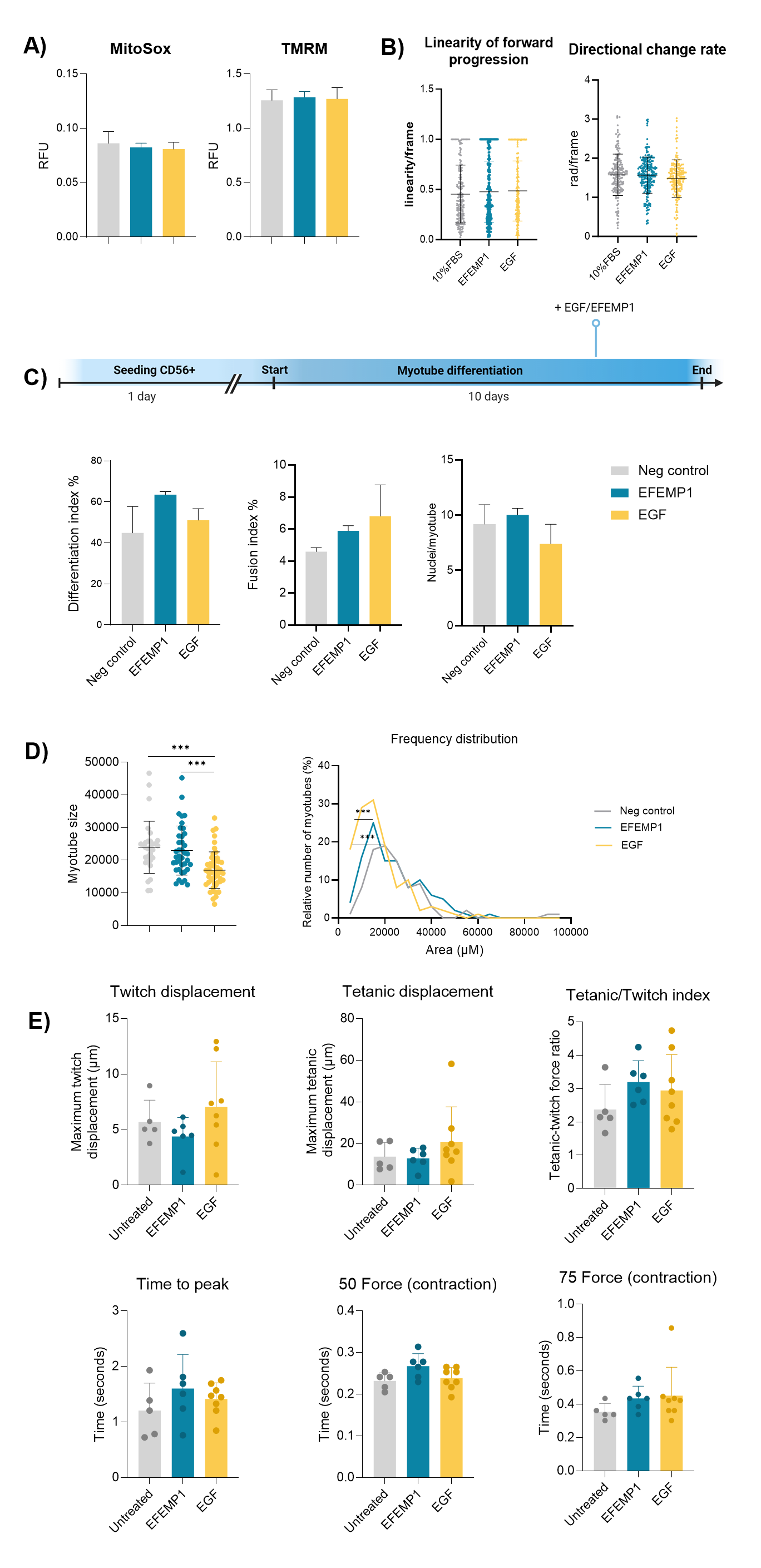

Supplement: Supplementary file 2 — Supplementary Figure 1 [file 41419_2025_8193_MOESM2_ESM.tif]
